# Environmental Variation Generates Environmental Opportunist Pathogen Outbreaks

**DOI:** 10.1371/journal.pone.0145511

**Published:** 2015-12-28

**Authors:** Jani Anttila, Veijo Kaitala, Jouni Laakso, Lasse Ruokolainen

**Affiliations:** Integrative Ecology Unit, Department of Biosciences, FI-00014 University of Helsinki, Helsinki, Finland; Université de Perpignan Via Domitia, FRANCE

## Abstract

Many socio-economically important pathogens persist and grow in the outside host environment and opportunistically invade host individuals. The environmental growth and opportunistic nature of these pathogens has received only little attention in epidemiology. Environmental reservoirs are, however, an important source of novel diseases. Thus, attempts to control these diseases require different approaches than in traditional epidemiology focusing on obligatory parasites. Conditions in the outside-host environment are prone to fluctuate over time. This variation is a potentially important driver of epidemiological dynamics and affect the evolution of novel diseases. Using a modelling approach combining the traditional SIRS models to environmental opportunist pathogens and environmental variability, we show that epidemiological dynamics of opportunist diseases are profoundly driven by the quality of environmental variability, such as the long-term predictability and magnitude of fluctuations. When comparing periodic and stochastic environmental factors, for a given variance, stochastic variation is more likely to cause outbreaks than periodic variation. This is due to the extreme values being further away from the mean. Moreover, the effects of variability depend on the underlying biology of the epidemiological system, and which part of the system is being affected. Variation in host susceptibility leads to more severe pathogen outbreaks than variation in pathogen growth rate in the environment. Positive correlation in variation on both targets can cancel the effect of variation altogether. Moreover, the severity of outbreaks is significantly reduced by increase in the duration of immunity. Uncovering these issues helps in understanding and controlling diseases caused by environmental pathogens.

## Introduction

Environmental opportunist pathogens are a large class of pathogens that have the ability to persist and grow in the outside host environment and invade the host under favourable conditions. The best-known example of an environmental opportunist human pathogen is *Vibrio cholerae*. In wildlife and livestock there are several economically important examples such as *Flavobacterium columnare* [1], *Bacillus anthracis* [2], and bat white-nose syndrome [3]. With free-living pathogens, the environment is a permanent source of new infections, which makes disease control challenging. Contrary to obligate pathogens, environmental opportunist diseases cannot be eradicated by treating hosts. Mathematical theory of infectious diseases has been originally centred on obligate pathogens emphasising direct transmission in host-to-host contacts [4]. More recently, there has been increasing interest in both empirical and theoretical studies on environmentally transmitted diseases [5,6], but so far only few articles have considered environmentally growing pathogens [7–10].

Many infectious diseases cause sporadic outbreaks or recurrent epidemics instead of being permanently prevalent. In classical epidemiology of obligate pathogens, the best-known examples are childhood diseases such as measles, influenza and common cold viruses. In the last decades, there has been increasing interest in similarly recurrent outbreaks of pathogens that have an environmental reservoir such as cholera [11] or avian influenza [12]. Much modelling effort has been put into explaining these outbreak patterns, and many factors have been identified to contribute to the frequency and intensity of outbreaks. For example environmental effects such as seasonality are clearly important [2,13–15]. Seasonal variation can also have important interactions with the immunisation process, which is an important factor in epidemiological dynamics [16]. Together with demographic turnover rate, the duration of immunity determines how quickly the susceptible host density is replenished after an outbreak, and consequently how frequent outbreaks can be. Immunity can be lost either due to waning immune response, as in pertussis [17], or pathogen mutations that reduce its recognition to the immune system, as in many viral diseases.

Environmental variation, which affects practically all natural systems, may be partly predictable due to temporal autocorrelation or periodicity, such as annual patterns in temperature and rainfall. However, an unpredictable component always remains, e.g. stochastic short-term fluctuations in the environmental variables. Environmental fluctuations are likely to play a role in various parts in ecological or epidemiological systems, affecting organism growth and mortality, or by modifying interactions [18,19]. While all pathogens can be affected by environmental variation indirectly within the host or during transmission, opportunist pathogens can spend several generations between infection events exposed to the outside host environment and as such their growth and transmission are directly subject to various abiotic and biotic pressures.

Opportunist pathogens that are aquatic, such as the fish pathogen *Flavobacterium columnare*, are heavily affected by water temperature [20], which has strong annual (periodic) component. Soil pathogens, such as *Bacillus anthracis*, are affected by rainfall in addition to temperature [2]. The potential importance of environmental drivers in eco-evolutionary dynamics of infectious diseases has been studied in vector-borne diseases [21], where environmental stochasticity has been recognised as potentially increasing the prevalence of disease and promoting virulence evolution. Recently, Anttila et al. [22] showed that the temporal structure of environmental fluctuations can have a qualitative impact on the nature of disease dynamics, such that either infection-free, persistent epidemic, and disease outbreak scenarios can arise.

Here we ask how environmental variation translates through the free-living environmental pathogen dynamics to changes in healthy host densities and epidemiology. The eco-epidemiological system can be seen as a filter for environmental variation [23]. Most work on environmental variation is so far limited to periodic variation, uncorrelated stochastic variation (i.e. white noise), or linear population dynamics [24], due to limitations of available analytical methods. Realistically complex models with temporally correlated stochasticity require numerical analysis and thus only a small portion of the full biologically relevant parameter space can be analysed. For generality, we inspect two important targets for periodic and stochastic environmental variation in our model: pathogen growth rate in the outside host environment, and 50% infective dose. The former reflects purely environmental effects on the pathogen proliferation, and the latter as an interaction parameter could be interpreted as changes in host immune system function, or changes in pathogen virulence. In either case, the temporal variation affects the propensity of contracting an infection from the environment, which can trigger pathogen outbreaks. The quality of variation determines the severity and frequency of outbreaks, but this is also affected by the expected duration of immunity, and whether the variation affects pathogen growth or immune system threshold.

## Materials and Methods

We constructed a model based on the classical SIRS-model where *S* denotes susceptible hosts, *I* infected hosts, and *R* recovered and immune hosts, which can return to the susceptible class *S* by loss of immunity. In addition, the model includes free-living pathogens, *P*.

$$\frac{dS}{dt}=r_{h}\left(S+R\right)-r_{h}S\frac{S+I+R}{K}-\beta Sf\left(P\right)+\rho R$$ (1.1)

$$\frac{dI}{dt}=\beta Sf\left(P\right)-r_{h}I\frac{S+I+R}{K}-\nu I-\delta I$$ (1.2)

$$\frac{dR}{dt}=\delta I-\rho R-r_{h}R\frac{S+I+R}{K}$$ (1.3)

$$\frac{dP}{dt}=r_{p}P\left(1+\theta_{1}\right)-\mu P^{2}+\lambda I+k\nu I$$ (1.4)

Susceptible hosts grow with rate *r*_*h*_. Both *S* and *R* contribute to the growth and are reduced by the density dependence, i.e. competition for limited resources defined by host carrying capacity *K*. Infected hosts compete for resources but do not contribute to population growth. We consider the infection severe enough to prevent reproductive effort and cause some mortality. Susceptible hosts can be infected by the free living pathogen depending on their densities and a maximum infection rate *β*. Infected hosts either recover with rate *δ* or die with rate *ν*. Recovered hosts are immune to the pathogen until they return to the susceptible class with rate *ρ*. The free-living pathogen grows with rate *r*_*p*_ and is limited by density dependent mortality rate *μ*. In addition pathogens are continuously shed from infected hosts with rate *λ* and released with host deaths with burst size *k*.

The infectivity functional response *f*(*P*) is assumed to be a sigmoidal function of the pathogen density:

$$f\left(P\right)=\frac{{\left(\frac{P}{(1+\theta_{2}){ID}_{50}}\right)}^{\kappa }}{1+{\left(\frac{P}{(1+\theta_{2}){ID}_{50}}\right)}^{\kappa }}$$ (2)

The sigmoidal shape of the infectivity response imposes an Allee effect on the pathogens: with densities under the infective dose *ID*_50_, the pathogens are less likely to cause infections. This is a realistic assumption for environmental pathogen—host infection process since it takes into account co-operative effort that many bacterial pathogens exhibit against the immune system, such as biofilm formation [25,26] and expression of virulence factors only in sufficient bacterial densities [27]. The sigmoidal infectivity response protects the host from infections by minute bacterial densities constantly encountered in the environment. The steepness of the sigmoidal curve is controlled by shape parameter *κ*. This form of infectivity response has been studied by Regoes et al. [28] in obligate pathogen context and by Anttila et al. [8,22] in an opportunistic pathogen context. The general behaviour of a similar model with environmental opportunist pathogens but without immunity or environmental variation was studied in [8], whereas in [22], the role of environmental stochasticity in facilitating pathogen emergence was studied.

Environmental variation is directed multiplicatively to either pathogen growth rate *r*_*p*_ or infectivity parameter *ID*_50_. These are denoted by *θ*_1_ and *θ*_2_, respectively. Both periodic annual variation and stochastic variation are considered. The periodic variation is assumed to be sinusoidal:

$$\theta \left(t\right)=A_{periodic}Z\;\left[\text{sin}\;\left(\frac{2\pi t}{365}\right)\right].$$ (3)

That is, an annual cycle of 365 days, which is commonly the most relevant periodic component in environmental conditions, daily period often being too rapid and others (such as lunar) being in most cases too weak to affect the system. Stochastic variation is generated using a 1/*f* noise generation procedure [29] to allow for different temporal structures, i.e., spectral “colours” of the noise [30]:

$$\theta \left(t\right)=A_{stochastic}Z\;\left[\sum_{j=1}^{n/2}\frac{1}{j^{-\gamma /2}}\text{sin}\;\left(\frac{2\pi t}{n}j+\varepsilon_{j}\right)\right]\;,$$ (4)

where *n* is the length of generated time series, *γ* is the spectral exponent [29], and *ε*_j_ are i.i.d-. uniform random numbers between zero and 2π (a random phase operator for each frequency *j*). The Z-function denotes a normalisation to zero mean and unit variance. In practice, environmental variations are generated separately, normalised to zero mean and unit variance, and then multiplied by a factor (*A*_periodic_)^1/2^ or (*A*_stochastic_)^1/2^ to obtain the desired variance. The variation is introduced to the continuous time differential equations by drawing a new value for the environmental condition for each day, effectively treating time *t* as an integer variable ranging from 1 to *n*. The drawn value is then held constant for the simulation steps within a day of simulated time. The spectral exponent *γ* is the slope of power spectrum of the generated noise time-series (monotonically related to autocorrelation on the first lag), i.e. controls the weights of faster and slower fluctuations in the stochastic variation. With *γ* = 0 the generated variation has zero autocorrelation (white noise). Decreasing *γ* gives more weight to slow fluctuations, and thus causes positive autocorrelation (red noise). Similarly, increasing *γ* gives more weight to fast fluctuations and thus results in negative autocorrelation (blue noise). The 1/f noise generation procedure tends to produce values that are not normally distributed when *γ* is decreased [31]. For this reason, we used a spectral mimicry method [32] to ensure that the noise frequency distribution is not affected by varying *γ*.

The simulated trajectories of the system were obtained by using a (fixed-step) Adams-Bashforth two-step routine in MATLAB (R2014b, Mathworks). The length of each generated time series was 8192 days. In addition, a deterministic version of the model was analysed with a continuer routine written in FORTRAN. In the latter case, the variances *A*_periodic_ and *A*_stochastic_ were set to zero.

Outbreaks were analysed from the simulated time series by discarding transients, collecting state variable minima, maxima, and means, and detecting any peaks that exceeded threshold values of *I* > 0.1*K in height and 50 days in duration. For each detected peak, we calculated the cumulative incidence as an integral over the number of infections within an outbreak: *cumulative incidence* = ∫ *βSf*(*P*) *dt*. The cumulative incidence is used as an indicator of outbreak severity. In addition, the healthy host (*S* + *R*) minimum is considered an indicator of overall risk of the disease eradicating the host.

The simulation parameters were selected such that the model system is approximately representative of a small fish host and a bacterial pathogen system (Table 1). The numbers of pathogens were scaled down by a factor of 10^6^ to obtain orders of magnitude similar to host densities. The infectivity parameters were chosen such that without environmental variation pathogen outbreaks do not occur and the number of infected hosts is negligible. This enables us to inspect the role of variation as a causative agent behind infections leading to pathogen outbreaks. We selected rate of immunity loss *ρ*, and environmental variation parameters *γ* and *A* as the parameters of interest for further analyses. The rate of immunity loss is important from the pathogen viewpoint since it determines how fast the supply of susceptible hosts is replenished after an outbreak (assuming *ρ* is higher than host growth rate). For simplicity, three specific values of immunity removal rate, ranging from long to rapid removal, were investigated: *ρ* = 0.01 d^–1^, *ρ* = 0.1 d^–1^, and *ρ* = 1.0 d^–1^. In the results and discussion sections these are referred to as long immunity, average immunity and short immunity, respectively. The expected duration of long immunity, 100 days is in the same magnitude as expected host generation length. The expected durations of average and short immunities are 10 and 1 days, respectively. The parameters *A* and *γ* affect the variance and frequency distribution of environmental variation.

**Table 1:** Model parameters and values used in simulations.

Symbol	Parameter name	Default value	Unit
*r*_*h*_	Host growth rate	0.01	*day* ^–1^
*K*	Host carrying capacity	100	*host*
*ρ*	Immunity loss rate	0.01, 0.1, 1.0	*day* ^–1^
*ν*	Infection mortality rate	0.02	*day* ^–1^
*δ*	Recovery rate	0.1	*day* ^–1^
*r*_*p*_	Pathogen growth rate	1.0	*day* ^–1^
*μ*	Pathogen mortality rate	0.002	(10^6^ *pathogens*)^–1^ *day* ^–1^
*λ*	Continuous shedding rate	80	10^6^ *pathogens host* ^–1^ *day* ^–1^
*k*	Burst size	300	10^6^ *pathogens host* ^–1^
*β*	Maximum infection rate	3.0	day^–1^
*ID*_50_	50% infective dose	2900	10^6^ *pathogens*
*κ*	Infectivity response shape parameter	5	–

To understand the results under environmental variation, the deterministic system was studied without environmental forcing. Here the hosts are effectively protected by the sigmoidal infectivity response, which reduces the rate of infection formation at low pathogen densities due to under-proportionately low infectivity. With the parameter values from Table 1 the equilibrium densities of *I* are low (*I* = 0.508). Increasing the pathogen growth rate *r*_p_ from 1.0 to 2.0 there is a region of steep increase in the equilibrium density of infected hosts, *I*, (S1 Fig) because of the sigmoidally shaped infectivity response (Eq 2). With higher maximum infectivity (*β* = 4.0) and short immunity (*ρ* = 1.0) part of this range (1.2 < *r*_p_ < 1.45) demonstrates cyclic dynamics (S2 Fig). A very small region of cyclic dynamics is retained when maximum infectivity is smaller (*β* = 3.0 and *ρ* = 1.0) but disappears quickly when immunity is longer. Adding a sufficient pathogen density as a pulse to the system or initiating the system with a sufficiently large pathogen density leads to an infection peak, the area of which is sigmoidally related to the pathogen density (S3 Fig).

## Results

A general pattern emerging from the results is that environmental variation affecting either pathogen growth rate *r*_p_ in the environmental reservoir or infective dose *ID*_50_ generally can cause pathogen outbreaks in a system where the disease has a low prevalence without variation. This validates the model as being capable of reproducing epidemiological dynamics observed in nature. When outbreaks are observed, increasing the expected duration of immunity significantly reduces their magnitude. This is because acquired immunity prevents re-infections of recovered hosts within an outbreak.

### Periodic vs. stochastic environmental variability

**Fig 1:**
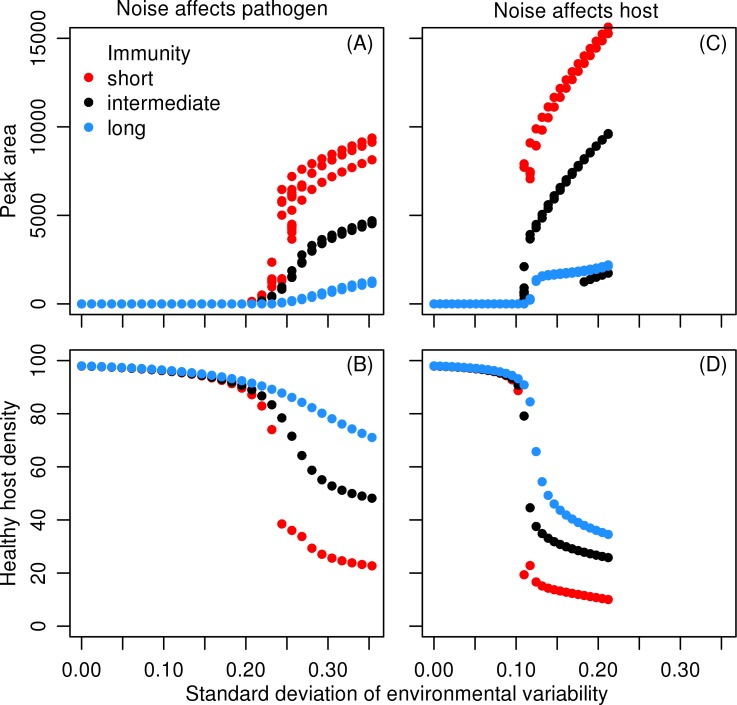
The effect of periodic variation. The effect of the variance of periodic environmental variation on the severity of outbreaks and healthy host density. Left panels (A) and (B): variations affect pathogen growth rate *r*_p_. Right panels (C) and (D): variations affect 50% infective dose *ID*_50_. Top panels (A) and (C) show the peak areas (outbreak severities). Bottom panels (B) and (D) show healthy host (S + R) density minima. Colours red, black, and blue show results on rapid, intermediate, and slow immunity loss rates, respectively. Under short immunity, interaction with a small cyclic range of pathogen growth rates (see Methods) causes three different peak areas per value, corresponding to three differently shaped infection cycles. See Table 1 for model parameters.

If fluctuations in the environment are periodic, disease outbreaks do not emerge unless the fluctuations are strong enough. This is due to the sigmoidal shape of infectivity response. Weak fluctuations have almost no effect on the system because they do not move the system across the infection threshold, arising from the sigmoidal infectivity function. When environmental variation affects pathogen growth rate, the threshold for outbreaks with the chosen parameter set is *A*_periodic_ ≈ 0.05 (Fig 1), and when 50% infective dose is affected, the outbreak threshold is roughly half of that in the previous case, *A*_periodic_ ≈ 0.015 (Fig 1). The epidemiological system is more sensitive to changes in *ID*_50_ since the infection process is much more sensitive to this parameter than to pathogen growth rate *r*_p_. A decrease in *ID*_50_ can immediately lead to infections and large inputs of pathogens to the environment. In contrast, increase in *ID*_50_ can immediately prevent infections.

**Fig 2:**
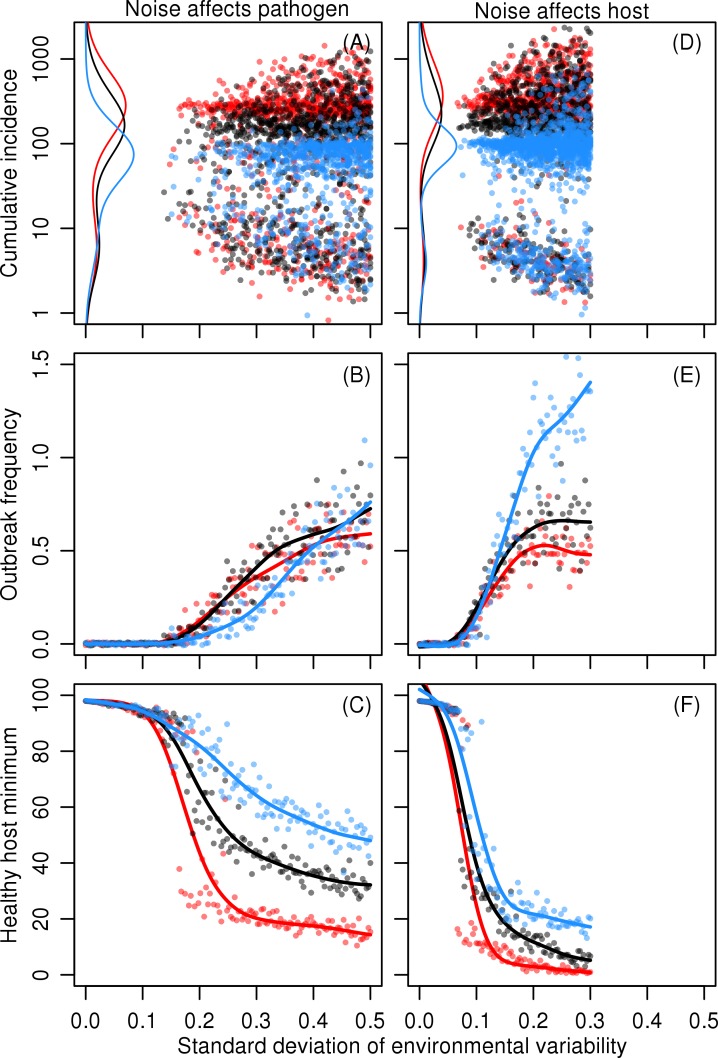
The effect of stochastic variation strength. The effect of pink stochastic variation on outbreak dynamics at different environmental variances. Left panels (A)–(C): variations affect pathogen growth rate *r*_p_. Right panels (D)–(F): variation affects 50% infective dose *ID*_50_. Top panels (A) and (D): cumulative incidences. Each dot represents a single outbreak, the number of which varies between simulations. The lines show a marginal kernel density estimate of peak areas. Panels (B), (C), (E) and (F): number of peaks and minimum values of healthy host density. Each dot represents a single simulation run. The lines show a smoothing spline fit to the points. In all simulations the spectral exponent *γ* = 1, i.e. pink noise. Colours red, black, and blue show results on rapid, intermediate, and slow immunity loss rates, respectively. See Table 1 for model parameters.

When periodic and stochastic environmental fluctuations have been scaled to have the same variance, much weaker stochastic forcing suffices to induce pathogen outbreaks, as compared to periodic forcing. This is because the range of values in periodic variation is bounded to standard deviation multiplied by square root of two, while 7.8% of normally distributed random values are more extreme than this value. Extreme values are highly important because the sigmoidal infectivity response gives an over-proportionate increase in infectivity with increasing pathogen density when the densities are low. From this perspective it is understandable that when stochastic variation affects pathogen growth rate, increasing noise variance increases the frequency of infection peaks (Fig 2).

The severity of disease outbreaks increases with both increasing noise variance and decreasing duration of immunity (Fig 2). The latter effect is due to two factors: the resistant individuals return to the susceptible class more rapidly, which fuels the build-up of the next outbreak, and the recovered individuals can be re-infected within the same outbreak. This leads to a longer duration of outbreaks, which is associated with increased severity and decreased frequency. Increasing noise variance also shows clearly that the distribution of outbreak severities is bimodal. This is due to the distribution of favourable periods to the pathogen. When the environmental variation produces a long favourable period, this always results in a severe outbreak that consumes the mass of healthy hosts and eventually subsides. However, shorter favourable periods occur more often, leading to a large number of less severe outbreaks.

### The temporal structure of stochastic variation

**Fig 3:**
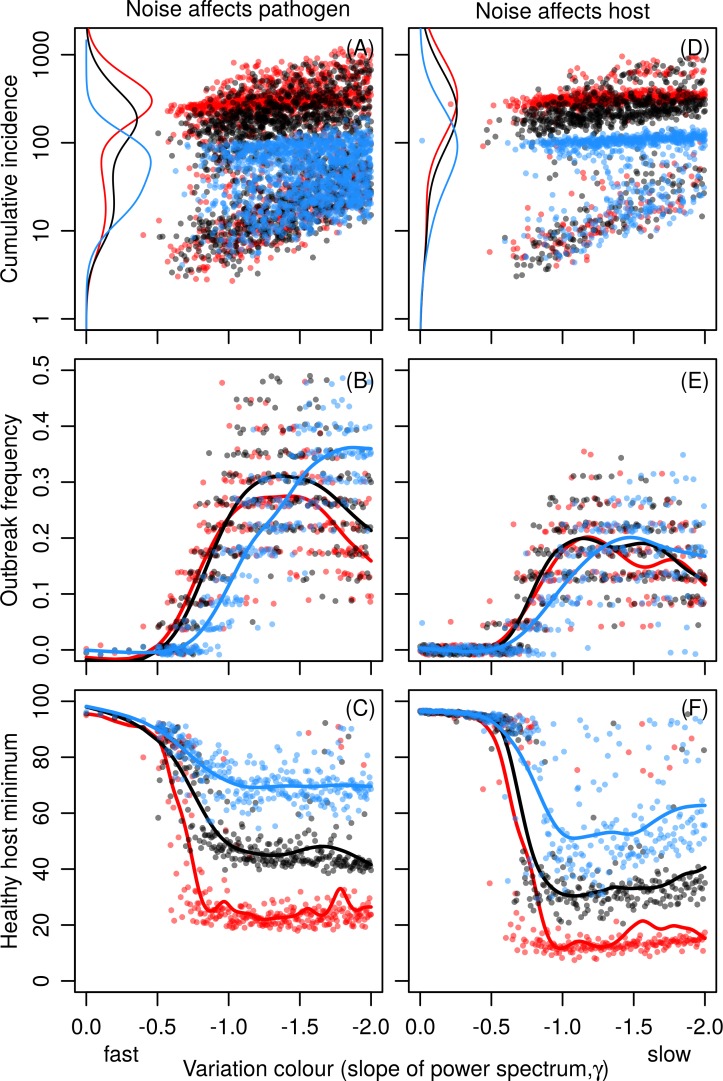
The effect of stochastic variation colour. The effect of the colour of stochastic variation (spectral exponent) on epidemiology. Left panels (A)–(C): variations affect pathogen growth rate *r*_p_. Right panels (D)–(F): variations affect 50% infective dose *ID*_50_. Top panels (A) and (D): Cumulative incidences. Each dot represents a single outbreak, the number of which varies between simulations, and the lines show a marginal kernel density estimate of peak areas. Panels (B), (C), (E) and (F): number of peaks and minimum value of healthy host density. Each dot represents a single simulation run. The lines show smoothing spline fits to the points. In the left panels variance *A* = 0.0625. In the right panels variance *A* = 0.01. Colours red, black, and blue show results on rapid, intermediate, and slow immunity loss rates, respectively. See Table 1 for model parameters.

In addition to variance, the effect of environmental variation is dependent on the temporal structure of the variation. Under weak forcing, deviations from the mean need to last long enough to cause an outbreak. Fast fluctuations, i.e., high frequency in the cyclic case and flat spectral density (i.e., low exponent *γ*, ‘white’ noise) in the stochastic case, are averaged out by the system and thus have little effect on the epidemiological dynamics (Fig 3). Environmental reddening (increasing the exponent *γ*) increases the effect of environmental variation to the system, which in turn increases the number and severity of pathogen outbreaks (Fig 3). However, outbreak frequency tends to peak at intermediate values of *γ* (e.g. pink noise), except under long immunity when forcing affects pathogen growth rate. The reason for this is that while extreme deviations from the mean are longer in duration under high values of *γ*, they occur less frequently than with intermediate values of *γ*. Intermediate values of *γ* cause deviations from the mean long enough to have an effect on the dynamical system while still varying relatively frequently.

### Environmental correlation between the pathogen and the host affects epidemiology

**Fig 4:**
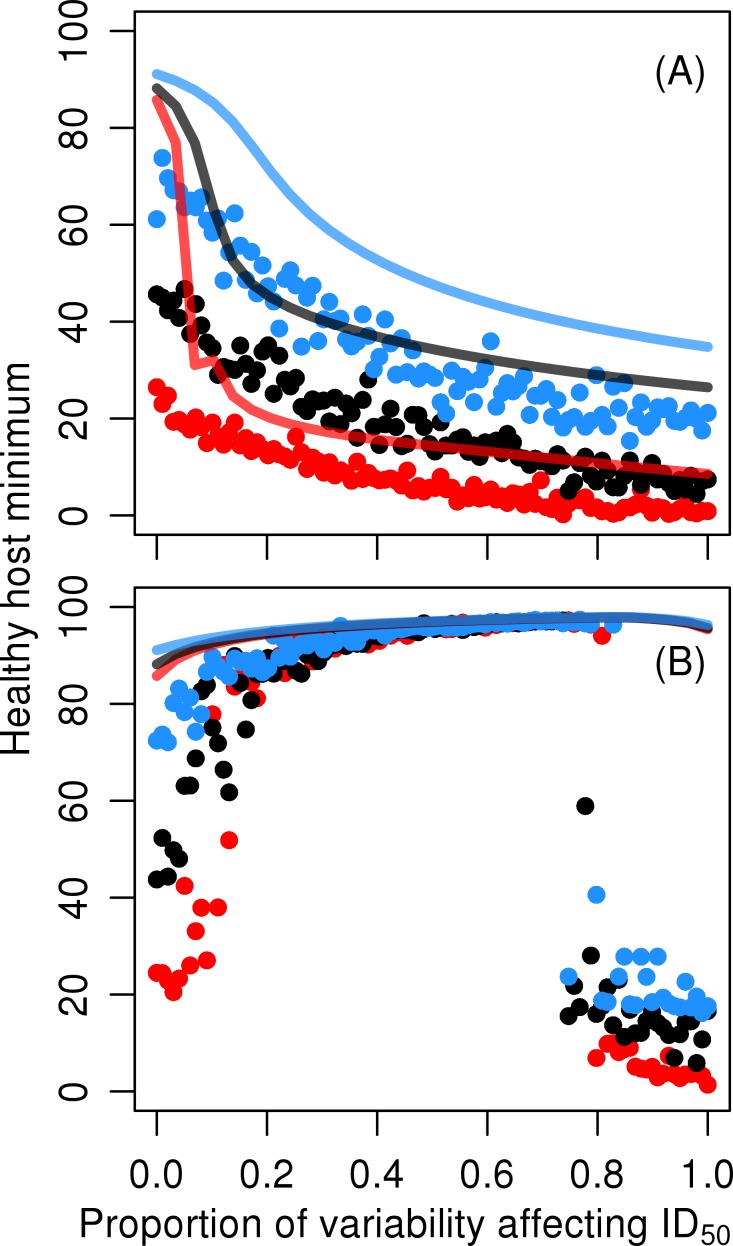
The effect of correlated variations. Effects of two stochastic environmental variations directed at pathogen growth rate *r*_p_ and 50% infective dose (*ID*_50_) in negative (A), and positive (B) correlation. X-axis shows relative variances of the two variations. Temporal colour of stochastic environmental variation was set to *γ* = –1, i.e. pink noise. The environmental variance directed to pathogen growth rate was set to *A*_1_ = 0.09. Line and dot colours red, black, and blue show results on rapid, intermediate, and long immunity loss rates, respectively. Each dot is a result of an individual stochastic simulation, while the lines present results from simulations with periodic forcing. See Table 1 for model parameters.

When environmental fluctuations simultaneously affect pathogen growth rate *r*_p_ and 50% infective dose *ID*_50_, the outcome depends on the relative variances and correlation between the fluctuations (Fig 4). Negatively correlated fluctuations generate more severe outbreaks since high pathogen growth rates coincide with low doses required for infection (Fig 4A). Under positively correlated fluctuations the effects of varying *r*_p_ and *ID*_50_ cancel out, unless the variation in *ID*_50_ is either much weaker or of comparable magnitude to that in *r*_p_. Periodic variations cancel each other out more completely because there are no random fluctuations. Stochastic variation can still cause extremes that sometimes result in outbreaks. However, only when the variation in *ID*_50_ is weak compared to that in *r*_p_, does the duration of immunity have a clear effect on outbreak severity. The intermediate case with independent variation in *r*_p_ and *ID*_50_ gives qualitatively similar results to those under negatively correlated fluctuations, so this scenario is not considered here further.

## Discussion

The central role of environmental variation in many ecological and epidemiological phenomena has been well recognised [18,30,33,34]. However, less attention has been paid to the role of the quality of environmental fluctuations in modifying epidemiological dynamics [16,35]: existing work has assumed overly simplistic periodic variation (but see [36]). To our knowledge, there are only few epidemiological studies that would acknowledge the potential importance of stochastic environmental fluctuations [21]. Here, we studied the role of such fluctuations in environmental opportunist pathogen outbreaks. These pathogens are naturally heavily influenced by environmental conditions since they depend on the free-living state for considerable duration between re-infecting hosts. Here we show that environmental variation is an important driver in epidemiological dynamics. Outbreaks can arise, given that the environmental amplitude is sufficiently high to cross the infective threshold and that fluctuations have a sufficiently low characteristic frequency, so that it is not averaged out by the system. An important feature is that reducing the expected duration of host immunity leads to more severe and longer outbreaks.

Environmental variation (periodic or stochastic), directed to pathogen growth rate, causes periods of elevated environmental pathogen densities, which can lead to disease outbreaks. The sigmoidal infectivity response assumed here results in a non-linear effect of variance (S3 Fig); as described above, outbreaks require an infection threshold to be crossed. Under periodic variation this trivially depends on the wave amplitude and less trivially on wave shape (the latter not investigated here). The effects of periodic and stochastic variation are for the most part similar and thus the periodic environmental variation gives a good approximation of how environmental variation affects an epidemiological system. However, stochastic variation causes more severe outbreaks. This means that, for a given variance, periodic variation underestimates the severity of outbreaks.

With stochastic environmental variation the frequency distribution determines how often the favourable environmental changes are persistent enough for the pathogen to reach significant densities. Fast variations (white noise, *γ* = 0) have relatively little effect on the dynamical system since the increases in pathogen growth rate (or decreases in 50% infective dose) are transient and are averaged out across time [37]. Moderately slow variations (pink noise, *γ* = 1) represent the most realistic proxy for natural environmental variation [38]. Interestingly, the lowest healthy host minima are achieved around this point, representing the largest risk to host population. Slow variations (brown noise, *γ* = 2) can result in more severe, but generally fewer outbreaks.

The frequency and duration of outbreaks is strongly affected by the formation of immunity and rate of immunity removal, which have important consequences on model dynamics. If the expected duration of immunity is short, the number of infections in an infection peak can exceed the host carrying capacity. This means that a disease can resemble an endemic state with recovered host reinfections sustaining the outbreak as long as the environment stays favourable for the pathogen. In general, long-term immunity effectively protects the hosts from severe outbreaks by preventing recovered host reinfections within an outbreak. Despite the reduced severity of outbreaks, the number of outbreaks can be larger than under intermediate or short durations of immunity.

While some infectious diseases lead to a long (pertussis) [39] or even lifelong (measles) [40] immunity, for many diseases the period of immunity after recovery is short or incomplete (RSV) [41]. Models assuming transient immunity can produce cyclic dynamics through Hopf bifurcations [42,43]. In addition, transient immunity further complicates disease control since a single control effort will not be sufficient [44]. Waning and imperfect immunities were studied by Gomes et al. [16] in obligate pathogen context. Their model contained a rate of immunity loss parameter that effectively controls the degree between SIR and SIS dynamics. Increasing the expected duration of immunity decreases the proportion of infected hosts and reduces the potential for oscillatory dynamics. In contrast, our model is stabilised by increasing the expected duration of immunity. This occurs because with shorter immunity the effect of infection mortality, a factor not considered by Gomes et al., is much larger, leading to depletion of hosts.

The results show that the sensitivity of different model parameters to environmental variation can have important effects on epidemiological dynamics. This has been recognised previously by Rohani et al. [36] who studied the effect of stochastic environmental variation on dynamics of childhood diseases. They discovered that measles outbreaks can be explained by purely deterministic dynamics whereas whooping cough outbreaks are only explained by adding environmental stochasticity to the model, because of differing infectious periods, despite their otherwise seemingly similar epidemiology. In our model the effect of environmental variation is generally stronger if it affects the 50% infective dose (*ID*_50_) than when the pathogen growth rate *r*_p_ varies. This is because decreases in *ID*_50_ are more directly translated to infections and subsequent pathogen output from hosts. However, in case the environmental stochasticity affects the pathogen growth rate *r*_p_ the average outbreak duration is longer. This is due to the effect of temporarily high growth rate affecting the system longer, until the pathogen density is reduced by competition, than temporarily low infective dose, which is instantly changed by environmental variation.

It follows that, if the two parameters (*ID*_50_ and *r*_p_) vary simultaneously, the correlation and relative magnitude of the fluctuations can become important to model dynamics, which is indeed the case here (Fig 4). An important finding is that periodic variation fails to qualitatively predict patterns in host dynamics when variation in the two parameters is correlated, where the relative magnitude of fluctuations can strongly affect host viability under stochastic forcing (Fig 4B). The periodic variation differs from stochastic variation in that for a given variance, the extreme values generated by stochastic variation can be much farther from the mean than the bounded extremes in periodic variation. The effect of simultaneous variation in several model parameters has not been previously considered in epidemiological systems and is rarely done even in ecological models (e.g. [19]).

Moreover, an eco-epidemiological system is often affected by different sources of environmental variation. The processes can be affected by the same or different sources of environmental variation, to varying degrees of correlation. In systems where environmental variation affects pathogen growth and host immune defences in positive correlation, these two effects can cancel each other, unless one of the effects is much stronger. Such a scenario is common in many terrestrial ecosystems since moderate increases in temperature lead to increase in both pathogen growth and immune system function. On the other hand, a scenario where pathogen growth and host immune defences are negatively correlated occurs in fisheries especially in salmonids, which tend to develop stress from temperature increases that enable bacterial growth in the spring [45,46]. This makes such systems more vulnerable to environmental variation altogether. In some cases the effects to pathogen growth and host immune function are mostly uncorrelated, e.g. systems in which immune defence is affected by temperature whereas pathogen growth is mostly limited by rainfall [2].

## Conclusions

Global warming is expected to increase the amplitude of environmental variance, and thus promote outbreaks, as well as making variations faster [47]. Also, short-term temporal structure of environmental fluctuations can vary considerably over time [18]. Dynamical systems typically have resonant or characteristic frequencies at which an external forcing amplifies any cyclic behaviour. The resonances of a classical SIR model have been investigated by Greenman et al. [35] and can be used to understand sub-harmonics that appear in, e.g. measles epidemics. The complex structure of temporal variation in environmental conditions, and the often very unpredictable behaviour of complex biological systems under perturbations, call for a better understanding of how stochastic forcing might impact on various systems. Here we have shown that the effects of environmental variability are profoundly modified by the type of environmental variability, and the underlying assumptions on the host immunity. Importantly, the temporal structure of the environmental variability is important: the often-found slow “pink” variation for example produced the highest risk for outbreaks.

## Supporting Information

S1 Fig Deterministic system. Infected (A) and healthy (*S* + *R*, B) host equilibrium densities on different pathogen growth rates without environmental variation. Parameters are set as in Table 1, with immunity loss rate *ρ* = 0.1. Panels (C) and (D) show Jacobian matrix eigenvalue real parts and imaginary parts, respectively.
(TIF)

S2 Fig Deterministic system with higher maximum infectivity. Infected (A) and healthy (*S* + *R*, B) host equilibrium densities on different pathogen growth rates without environmental variation. Here maximum infectivity rate is higher (*β* = 4.0). Otherwise parameters are set as in Table 1, with immunity loss rate *ρ* = 1.0. Panels (C) and (D) show Jacobian matrix eigenvalue real parts and imaginary parts, respectively.
(TIF)

S3 Fig Infection peak as a response to pathogen pulse. Outbreak severities (peak areas) resulting from pulsed pathogen input to the system at an equilibrium. Parameters are set as in Table 1 (main text). Expected duration of immunity is set to average (*ρ* = 0.1).
(TIF)

S4 Fig Example stochastic time series. Top panel (A) shows infected (red), immunised (green), susceptible (black), and healthy (grey) host densities. Bottom panel (B) shows pathogen densities (black) and the value of environmental stochasticity (grey) scaled to the centre of the panel. Here the environmental stochasticity is directed at pathogen growth rate *r*_p_. Parameters are set as in Table 1 (main text). Expected duration of immunity is set to average (*ρ* = 0.1).
(TIF)

S1 Text Analysis of deterministic system and example time-series. (DOCX)
