# Supplementary figures and images for: Environmental Variation Generates Environmental Opportunist Pathogen Outbreaks
Source: PLoS One. 2015 Dec 28;10(12):e0145511. doi: 10.1371/journal.pone.0145511 (PMC4692394; doi:10.1371/journal.pone.0145511)

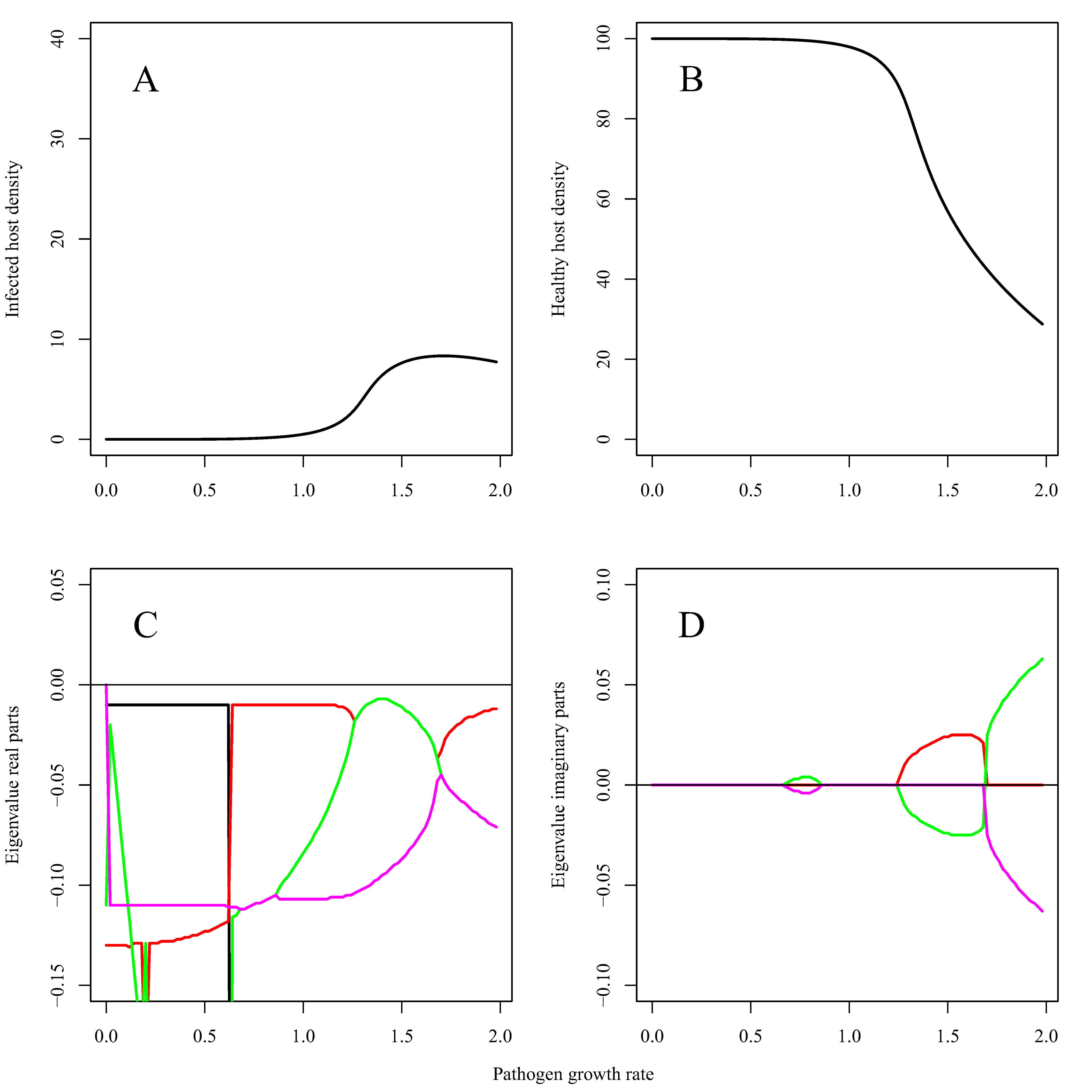

Supplement: S1 Fig — Infected (A) and healthy (S + R, B) host equilibrium densities on different pathogen growth rates without environmental variation. Parameters are set as in Table 1, with immunity loss rate ρ = 0.1. Panels (C) and (D) show Jacobian matrix eigenvalue real parts and imaginary parts, respectively. (TIF) [file pone.0145511.s001.tif]

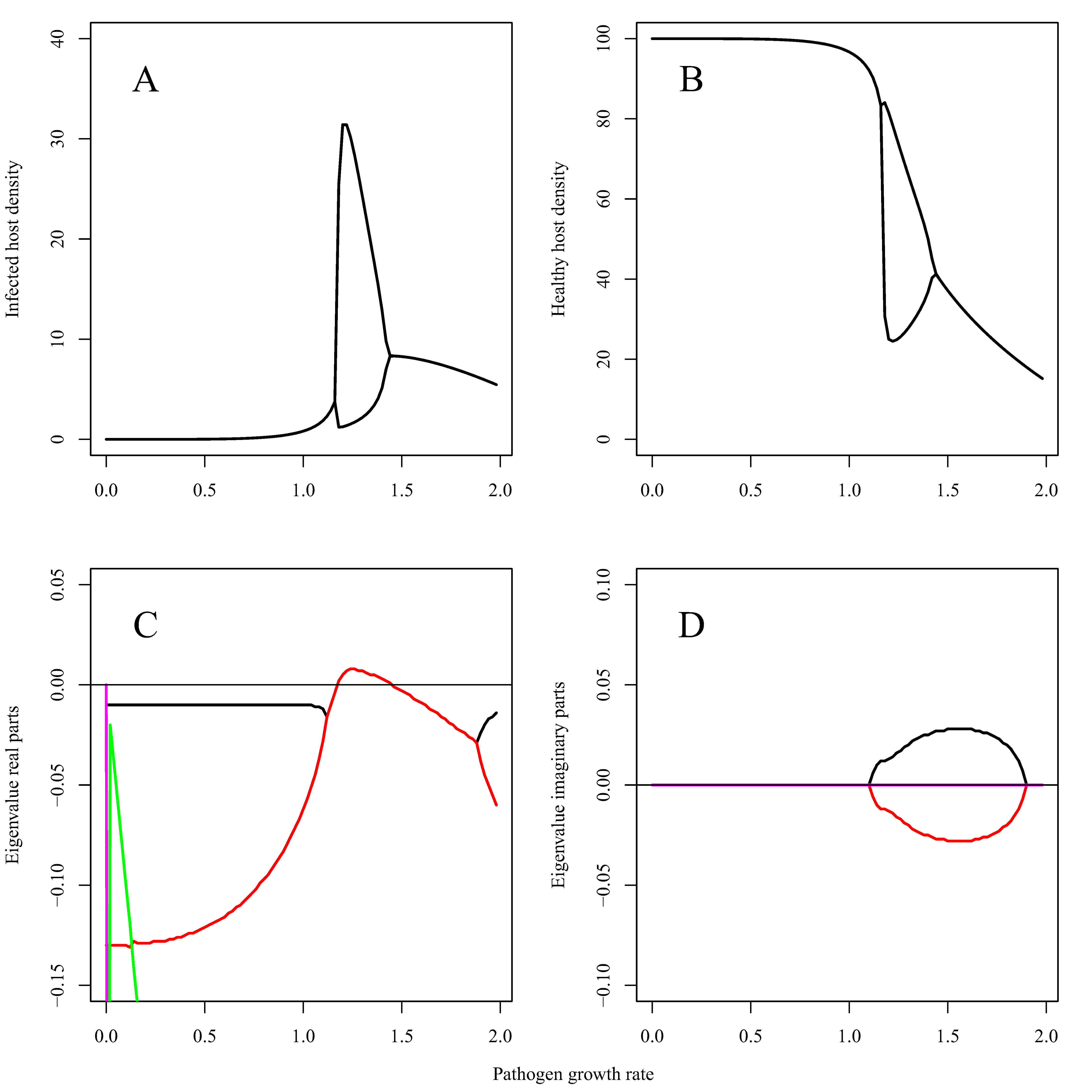

Supplement: S2 Fig — Infected (A) and healthy (S + R, B) host equilibrium densities on different pathogen growth rates without environmental variation. Here maximum infectivity rate is higher (β = 4.0). Otherwise parameters are set as in Table 1, with immunity loss rate ρ = 1.0. Panels (C) and (D) show Jacobian matrix eigenvalue real parts and imaginary parts, respectively. (TIF) [file pone.0145511.s002.tif]

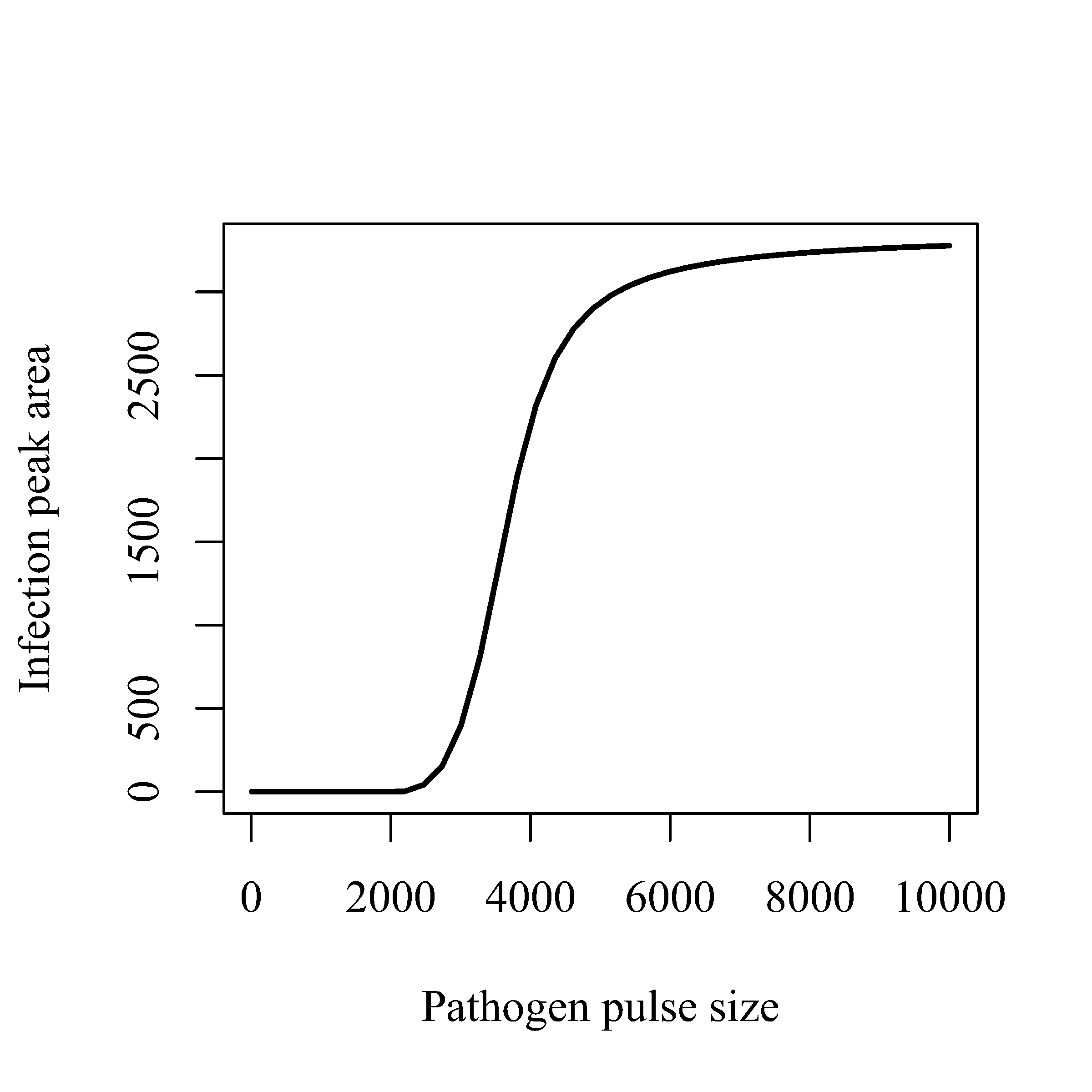

Supplement: S3 Fig — Outbreak severities (peak areas) resulting from pulsed pathogen input to the system at an equilibrium. Parameters are set as in Table 1 (main text). Expected duration of immunity is set to average (ρ = 0.1). (TIF) [file pone.0145511.s003.tif]

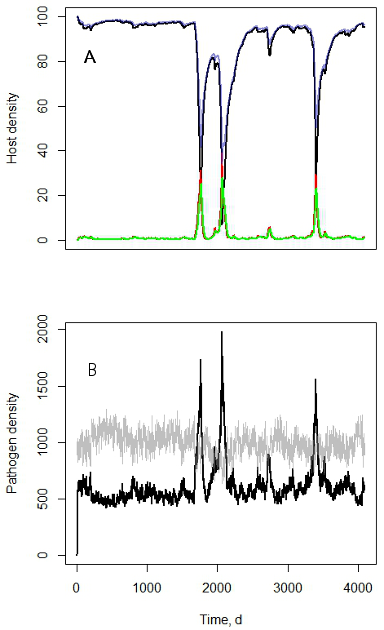

Supplement: S4 Fig — Top panel (A) shows infected (red), immunised (green), susceptible (black), and healthy (grey) host densities. Bottom panel (B) shows pathogen densities (black) and the value of environmental stochasticity (grey) scaled to the centre of the panel. Here the environmental stochasticity is directed at pathogen growth rate r p. Parameters are set as in Table 1 (main text). Expected duration of immunity is set to average (ρ = 0.1). (TIF) [file pone.0145511.s004.tif]
