# Supplementary material for: Environmental Variation Generates Environmental Opportunist Pathogen Outbreaks
Source: PLoS One. 2015 Dec 28;10(12):e0145511. doi: 10.1371/journal.pone.0145511 (PMC4692394; doi:10.1371/journal.pone.0145511)
Supplement: S1 Text — (DOCX) [file pone.0145511.s005.docx]

### Supporting Infromation

To demonstrate the effect of different pathogen growth rates *r*_p_, the deterministic system (eqs. 1 and 2, *θ*_1_ = 1.0, *θ*_2_ = 1.0) was simulated numerically with increasing *r*_p_ (S1 Fig). Because of the sigmoidal shape of infectivity response, the equilibrium density of infected, *I*, increases sigmoidally until it begins decreasing due to infection mortality. Assuming parameters values from Table 1 the equilibrium densities of the community are locally stable. If the maximum infectivity of the pathogen is higher (β=4.0) and the expected duration of immunity is short (ρ=1.0) the deterministic system undergoes a Hopf-bifurcation to cyclic dynamics when pathogen growth rate exceeds critical value *r*_p,c_ = 1.2 (S2 Fig).

Initiating the deterministic system with large initial pathogen densities (pathogen pulses) leads to infection peaks ($peak area=\int K_{h}-\left( S\left( t \right)+R\left( t \right) \right) dt$) that increase sigmoidally as the pulse size increases (S3 Fig). Similarly to this effect, environmental stochasticity can initiate sharp infection peaks by providing periods of increased pathogen growth *r*_p_ (S4 Fig) or decreased 50% infective dose *ID*_50_.
